# Predicting community acquired bloodstream infection in infants using full blood count parameters and C-reactive protein; a machine learning study

**DOI:** 10.1007/s00431-024-05441-6

**Published:** 2024-04-18

**Authors:** Lieke Brouwer, Robert Cunney, Richard J. Drew

**Affiliations:** 1 Public Health Laboratory, HSE, Cherry Orchard Hospital, Dublin, Ireland; 2 European Public Health Microbiology Training Programme (EUPHEM), European Centre for Disease Prevention and Control, Stockholm, Sweden; 3 Irish Meningitis and Sepsis Reference Laboratory, Children’s Health Ireland at Temple Street, Dublin, Ireland; 4 Department of Clinical Microbiology, Royal College of Surgeons in Ireland, Dublin, Ireland; 5 Clinical Innovation Unit, Rotunda Hospital, Dublin, Ireland

**Keywords:** Bacteraemia, Blood stream infection, Machine Learning, Predictive model

## Abstract

Early recognition of bloodstream infection (BSI) in infants can be difficult, as symptoms may be non-specific, and culture can take up to 48 h. As a result, many infants receive unneeded antibiotic treatment while awaiting the culture results. In this study, we aimed to develop a model that can reliably identify infants who do not have positive blood cultures (and, by extension, BSI) based on the full blood count (FBC) and C-reactive protein (CRP) values. Several models (i.e. multivariable logistic regression, linear discriminant analysis, K nearest neighbors, support vector machine, random forest model and decision tree) were trained using FBC and CRP values of 2693 infants aged 7 to 60 days with suspected BSI between 2005 and 2022 in a tertiary paediatric hospital in Dublin, Ireland. All models tested showed similar sensitivities (range 47% – 62%) and specificities (range 85%-95%). A trained decision tree and random forest model were applied to the full dataset and to a dataset containing infants with suspected BSI in 2023 and showed good segregation of a low-risk and high-risk group. Negative predictive values for these two models were high for the full dataset (> 99%) and for the 2023 dataset (> 97%), while positive predictive values were low in both dataset (4%–20%).

*Conclusion*: We identified several models that can predict positive blood cultures in infants with suspected BSI aged 7 to 60 days. Application of these models could prevent administration of antimicrobial treatment and burdensome diagnostics in infants who do not need them.

**Table:** 

**What is Known:** *• Bloodstream infection (BSI) in infants cause non-specific symptoms and may be difficult to diagnose.* *• Results of blood cultures can take up to 48 hours.*	
**What is New:** *• Machine learning models can contribute to clinical decision making on BSI in infants while blood culture results are not yet known.*

**Supplementary Information:**

The online version contains supplementary material available at 10.1007/s00431-024-05441-6.

## Introduction

Detection of bacteria from blood cultures in children may be due to bloodstream infection (BSI), transient (non-significant) bacteraemia, or contamination of the blood culture by skin flora. BSIs are a leading cause of morbidity and mortality in infants world-wide [1–3]. The main pathogens involved in BSIs in infants are Gram-negative bacteria (GNB) (e.g., *E. coli, Pseudomonas* spp. and *Klebsiella* spp.) and Group B streptococcus (GBS) [3–6].

Diagnosis of BSI by blood culture can take up to 48 h [7]. This can result in administration of unnecessary treatment or the execution of burdensome diagnostic procedures, such as lumbar puncture.

A combination of clinical signs, biomarkers, and full blood count (FBC) parameters is used for early recognition of BSI. However, in neonates and infants, these signs and markers can be difficult to interpret, as symptoms can ben non-specific and normal ranges of biomarkers and FBC parameters change during the first months of life [8]. Efforts have been put into deriving clinical algorithms that reliably predict BSI upon presentation based on these parameters [9, 10]. Though much progress has been made, the distinction between patients with and without BSI is still not perfect.

It has been suggested that machine learning could contribute to the development of algorithms with a higher accuracy of predicting BSI compared to existing methods [11]. In several patient groups, machine learning algorithms have already shown promising results in predicting of positive blood cultures (and, by extension, BSI) from clinical symptoms, biomarkers and FBC parameters [12, 13]. Some efforts have already been made to apply machine learning methods to predict BSI in infants; Ramgopal et al. showed that a random forest model can predict serious bacterial infections in infants with high specificity and sensitivity [14]. Application of such a model in clinical practice could prevent many infants without BSI having to undergo burdensome diagnostic procedures and unnecessary treatment.

The aim of our current study was to derive an easy-to-apply algorithm that can reliably identify infants with a low risk of community acquired BSI. To do this we applied various machine learning methods on FBC and CRP data from a cohort of infants aged 7 to 60 days presenting at the emergency department in a tertiary hospital in Dublin.

## Methods and materials

### Study population

We performed a retrospective case-control study at Children’s Health Ireland (CHI) Temple Street, Dublin, a tertiary paediatric hospital. The CHI Research Ethics Committee (REC) approved this study (reference number: REC-194-22). Our study population was composed of all infants aged 7 to 60 days who were admitted via the emergency department of CHI, Temple Street between January 1st 2005 and December 17th 2022, and received a work-up for suspected BSI upon presentation. All infants with a positive or negative blood culture, and who had an FBC and C-reactive protein (CRP) taken as part of their initial investigations, were included. The age range 7 to 60 days was chosen as during this period of life, the reference values for FBC parameters and CRP are relatively stable, while several reference values change considerably in the first days of life and after the first months of life. All Infants with a positive blood culture were included as cases, while all infants with a negative blood culture were included as controls. Infants that did not have FBC, CRP and blood culture results from samples taken on the same day were excluded from analysis. All blood cultures were processed in the microbiological laboratory at CHI, Temple Street using an automated blood culture platform (BacT/ALERT, BioMérieux, Marcy-l'Étoile, France), and this methodology did not change during the study period.

### Data collection

Data were extracted from the electronic laboratory information system at CHI Temple Street on the following parameters: age in days on date of sampling, sex, year of sampling, FBC parameters (i.e. white cell count (WCC), neutrophils (N), lymphocytes (L), monocytes (M), eosinophils (E), basophils (B), platelets (PLT), red cell count (RCC), red cell distribution width (RDW), haemoglobin (Hb), haematocrit (HCT), mean platelet volume (MPV), mean corpuscular haemoglobin (MCH), mean corpuscular haemoglobin concentration (MCHC) and mean corpuscular volume (MCV)), CRP and blood culture results. Ratios were calculated for neutrophiles to lymphocytes (NLR), MPV to platelets (MPVPR), monocytes to lymphocytes (MLR) and platelets to lymphocytes (PLR). As data on clinical parameters – e.g. temperature, heart rate and treatment regime – are stored in different databases which are not straightforward to merge with the laboratory databases, we were currently unable to incorporate clinical parameters in our analysis. Cases with blood culture results positive for organisms classified as likely contaminants (Supplementary Material 1) or fungal pathogens were excluded from the dataset, to ensure that included cases with positive blood cultures were likely to represent bacterial BSI. The cases were subsequently grouped into three discrete groups: Gram negative bacteraemia, bacteraemia with Group B Streptococcus, and other clinically significant bacteraemia.

### Data analysis

Cut-off values for FBC outliers were decided based on the distribution patterns of the FBC variables and clinical experience. Infants with outliers in any of the FBC variables that were likely to be derived from underlying conditions such as malignancies were excluded from further analysis. Infants that were missing CRP values, values for any FBC variable or data on sex or age were excluded from further analysis. Differences in baseline characteristics age and sex between the cases and controls were assessed by Chi-squared test and Student’s t-test respectively. Exploratory univariate and multivariate analyses were performed using the ggplot2 package [15] in R statistical software version 4.2.2 [16] to construct violin plots and a heatmap. To calculate the association between the independent variables age, sex, FBC parameters and CRP and the dependent variable blood culture result, univariable logistic regression was performed using the gtsummary package [17] in R statistical software.

To be able to train our models using reasonably balanced data, the controls in our dataset were randomly subsampled to reach a case control ratio of 1:3. The subsampled dataset was then randomly split into a training set containing 70% of the data and a test set containing 30% of the data. A multivariable logistic regression model was build based on the training set in R. The model was build based on the results of the univariable model, including all variables with a p-value of 0.2 or less, and subsequently removing variables using the top-down strategy to obtain a model in which all independent variables were significant (p-value < 0.05).

The training set was normalized, and a linear discriminant analysis (LDA) model was build based on the training set using the MASS package [18] in R. A K-nearest neighbour (KNN) model and a support vector machine (SVM) with linear kernel were fitted to the normalized training set using the class [18] and e1071 [19] packages respectively in R. A decision tree and a random forest were build based on the non-normalized training set using the rpart package [20] and randomForest package [21] respectively in R. All analyses were performed including variables with a p-value of 0.2 or less in the univariable regression model, to minimize excluding variables that would make a valuable contribution to the models, as well as to minimize including variables that would not be of importance and would create noise in the model. Additionally, an LDA model and decision tree model were build based on the grouping of pathogens (GNB versus GBS versus controls) in the training set.

All models were subsequently used to make predictions in the test set, and parameters for the prediction (sensitivity, specificity, negative predictive value (NPV), positive predictive value (PPV) and accuracy) were calculated using the epiR package [22]. For each model, the area under the receiver operating characteristic curve (AUROC) was calculated using the pROC package [23].

The decision tree model and the random forest model that were trained using the subsampled dataset were subsequently used to make predictions for the entire dataset (i.e. 76 cases and 2616 controls). Sensitivity, specificity, NPV and PPV were calculated.

### Applying the model to a 2023 dataset

After finalisation of the models, 206 infants between the age of 7 and 60 days with a work-up for suspected BSI at CHI Temple Street between January 1st 2023 and September 27th 2023 were included as a second cohort. A prediction was made on the presence of bacteraemia in these children using the trained decision tree model and random forest model. These predictions were subsequently compared to the outcome of the blood culture, and accuracy, sensitivity, specificity, NPV and PPV were calculated.

## Results

### Dataset construction and descriptive analyses

In total, 2876 infants between 7 and 60 days of age presenting between 2005 and 2022 at the emergency department of CHI Temple Street, had received a work-up for suspected BSI. For 179 (6.2%) infants, the blood culture grew a likely contaminant, and these infants were subsequently excluded from analysis. A further 4 (0.1%) infants were excluded as their FBC results contained outliers. One infant (0.03%) was excluded as their sex was reported as unknown.

Of the remaining 2692 infants, 76 (2.9%) had a positive blood culture and were thus labelled cases, while 2617 (97.1%) had a negative blood culture and were thus labelled controls.

**Fig. 1:**
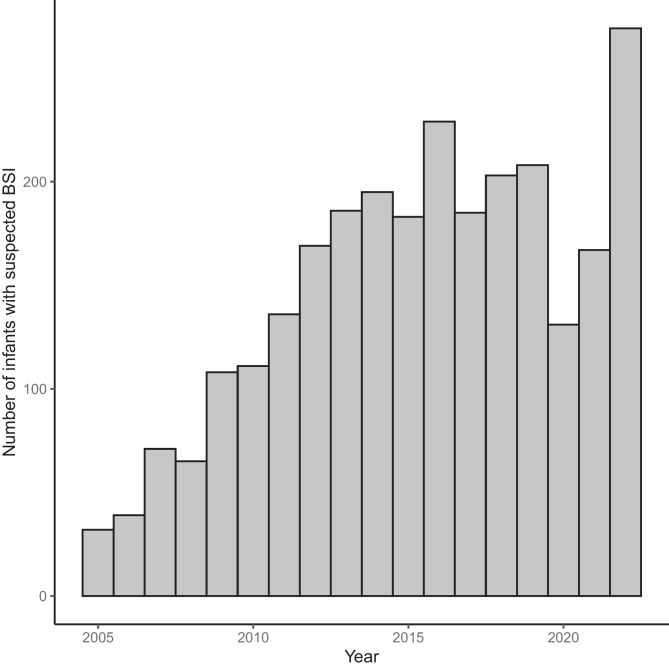
Histogram showing the annual number of infants aged 7–60 days assessed for suspected blood stream infection at Children’s Health Ireland Temple Street, Dublin, between 2005 and 2022

**Fig. 2:**
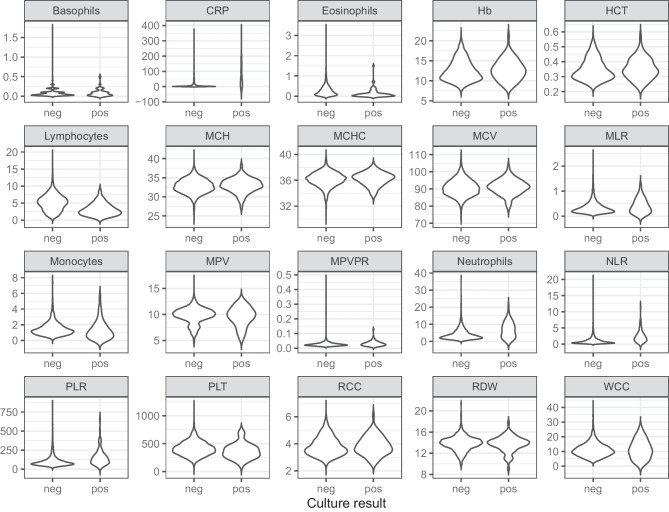
Violin plots showing the distribution of full blood count (FBC) parameters and C-reactive protein (CRP) for infants aged 7 – 60 days assessed for suspected bloodstream infection at Children’s Health Ireland Temple Street, Dublin, between 2005 and 2022. Results for children with positive (cases, N = 76) and negative (controls, N = 2616) blood culture results are shown. Basophils (10^9^/L), CRP; C-reactive protein (mg/L), Eosinophils (10^9^/L), Hb; haemoglobin (g/dL), HCT; haematocrit (L/L), Lymphocytes (10^9^/L), MCH; mean corpuscular haemoglobin (pg), MCHC; mean corpuscular haemoglobin concentration (g/dL), MCV; mean corpuscular volume (fL), Monocytes (10^9^/L), MPV; mean platelet volume (fL), Neutrophils (10^9^/L), PLT; platelets (10^9^/L), RCC; red cell count (10^12^/L), RDW; red cell distribution width (%), WCC; white cell count (10^9^/L), NLR; neutrophiles to lymphocytes ratio, MPVPR; MPV to platelets ratio, MLR; monocytes to lymphocytes ratio, PLR; platelets to lymphocytes ratio

**Fig. 3:**
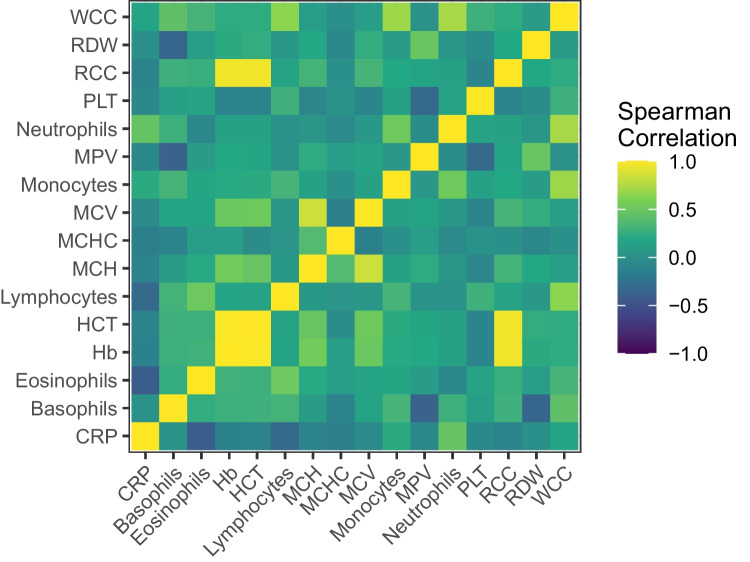
Heat plot showing the correlation between each of the full blood count (FBC) variables and C-reactive protein (CRP) in the full dataset (N = 2692) as calculated by the Spearman test. WCC, white cell count; RDW, red cell distribution width; RCC, red cell count; PLT, platelets; MPV, mean platelet volume; MCHC, mean corpuscular haemoglobin concentration; MCH, mean corpuscular haemoglobin; HCT, haematocrit; Hb, haemoglobin

Sex distribution was comparable between the case (34% female) and control group (44% female) (p = 0.11). The median age was 25 days for cases (inter quartile range (IQR) 17–39 days) and 34 days for controls (IQR 21 – 46 days) (p = 0.003). Between 32 and 274 infants were included in each calendar year, with lower numbers of inclusions during the earlier years in our study period, and during the first two years of the SARS-CoV-2 pandemic (2020–2021), while the highest number of inclusions was in 2022 (Fig. 1). Distribution of CRP and the FBC variables in cases and controls are shown in Fig. 2. The heat plot (Fig. 3) shows that WCC, Neutrophils and Lymphocytes were mutually correlated, as were RCC, Hb, HCT, MCV and MCH.

### Logistic regression models

In the univariable logistic regression analysis, age, CRP, E, L, N, PLT, NLR, MPVPR and PLR were significantly associated with blood culture results (p-value < 0.05) (Table 1).

**Table 1:** Univariable logistic regression performed on the full dataset (N = 2692). Odds ratios (OR) with 95% confidence intervals (CIs) and p-values are shown, and refer to the odds of having a positive blood culture result

**Characteristic**	**OR**^a^	**95% CI**^a^	**p-value**
Sex	1.51	0.94, 2.48	0.092
Age	0.98	0.96, 0.99	0.004
CRP	1.01	1.01, 1.02	< 0.001
Basophils	1.56	0.18, 6.04	0.6
Eosinophils	0.03	0.01, 0.13	< 0.001
Hb	1.03	0.94, 1.12	0.5
HCT	2.20	0.09, 46.4	0.6
Lymphocytes	0.64	0.57, 0.73	< 0.001
MCH	1.05	0.93, 1.19	0.4
MCHC	1.05	0.86, 1.29	0.6
MCV	1.01	0.97, 1.06	0.6
Monocytes	0.80	0.58, 1.07	0.2
MPV	0.93	0.81, 1.08	0.3
Neutrophils	1.16	1.10, 1.21	< 0.001
PLT	1.00	1.0, 1.00	0.002
RCC	1.08	0.78, 1.48	0.6
RDW	0.96	0.81, 1.14	0.6
WCC	1.02	0.97, 1.06	0.5
NLR	1.39	1.26, 1.53	< 0.001
MPVPR	1.13	0.99, 1.28	0.040
MLR	1.91	0.90, 3.62	0.067
PLR	1.00	1.00, 1.01	< 0.001

*CRP* C-reactive protein, *Hb* haemoglobin, *HCT* haematocrit, *MCH* mean corpuscular haemoglobin, *MCHC* mean corpuscular haemoglobin concentration, *MCV* mean corpuscular volume, *MPV* mean platelet volume, *PLT* platelets, *RCC* red cell count, *RDW* red cell distribution width, *WCC* white cell count, *NLR* neutrophiles to lymphocytes ratio, *MPVPR* MPV to platelets ratio, *MLR* monocytes to lymphocytes ratio, *PLR* platelets to lymphocytes ratio. Due to its small values, the MPVPR variable was scaled for this analysis

^a^*OR* Odds Ratio, *CI* Confidence Interval

Working top-down from a logistic regression model including all parameters with a p-value of 0.2 or lower in the univariable analysis, we derived a multivariable logistic regression model that included age, CRP, lymphocytes, NLR and MLR as significantly associated with the blood culture results.

### Predictive algorithms

The multivariable logistic regression, LDA, KNN, SVM, decision tree and random forest models were all able to predict bacteraemia from FBC parameters and CRP in infants in the subsampled test set with accuracies between 80 and 86%. All models showed moderate to high AUROCs (0.72 – 0.82), and high specificities (ranging between 85 and 95%), but rather low sensitivities, ranging from 47 to 61%. PPV was lowest for the decision tree model at 56.5%, while it was highest for the logistic regression model at with 75%. NPVs for all models were similar, ranging from 85 to 89% (Table 2). Across the multivariable logistic regression model, LDA, random forest and decision tree, the variables CRP, lymphocytes and NLR seemed particularly of importance.

**Table 2:** Area under the receiver operating characteristic curve (AUROC), accuracy (acc), sensitivity (sens), specificity (spec), positive predictive values (PPV) and negative predictive values (NPV) for the multivariable logistic regression model, linear discriminant analysis model, K-nearest neighbors model, support vector machine model, random forest model and decision tree model

**Dataset**	**Model**	**AUROC**	**CI AUROC**		**Acc (%)**	**CI Acc**		**Sens (%)**	**CI Sens**	
			lower	upper		lower	upper		lower	upper
Subsampled dataset	Multivariable Logistic Regression	0.82	0.70	0.93	85.9	77.0	92.3	57.1	34.0	78.2
(n = 304)	Linear Discriminant Analysis	0.74	0.61	0.87	81.5	72.1	88.9	47.6	25.7	70.2
	K-Nearest Neighbors	0.76	0.63	0.88	82.6	73.3	89.7	52.4	29.8	74.3
	Support Vector Machine	0.79	0.68	0.91	84.8	75.8	91.4	57.1	34.0	78.2
	Random Forest	0.81	0.69	0.92	84.8	75.8	91.4	57.1	34.0	78.2
	Decision Tree	0.72	0.61	0.84	80.4	70.9	88.0	61.9	38.4	81.9
Full dataset	Decision Tree	0.57	0.55	0.59	88.2	86.9	89.3	71.1	59.5	80.9
(n = 2692)	Random Forest	0.60	0.58	0.62	89.5	88.3	90.6	88.2	78.7	0.94
2023 Cohort	Decision Tree	0.58	0.50	0.66	52.3	45.1	59.4	57.1	18.4	90.1
(n = 197)	Random Forest	0.59	0.52	0.67	62.4	55.3	69.2	71.4	29.0	96.3

**Dataset**	**Model**	**Spec (%)**	**CI Spec**		**PPV (%)**	**CI PPV**		**NPV (%)**	**CI NPV**	
			lower	upper		lower	upper		lower	upper
Subsampled dataset	Multivariable Logistic Regression	94.4	86.2	98.4	75.0	47.6	92.7	88.2	78.7	94.4
(n = 304)	Linear Discriminant Analysis	91.5	82.5	96.8	62.5	35.4	84.8	85.5	75.6	92.5
	K-Nearest Neighbors	91.5	82.5	96.8	64.7	38.3	85.8	86.7	76.8	93.4
	Support Vector Machine	93.0	84.3	97.7	70.6	44.0	89.7	88.0	78.4	94.4
	Random Forest	93.0	84.3	97.7	70.6	44.0	89.7	88.0	78.4	94.4
	Decision Tree	85.9	75.6	93.0	56.5	34.5	76.8	88.4	78.4	94.9
Full dataset	Decision Tree	88.7	87.4	89.9	15.4	11.8	19.6	99.1	98.6	99.4
(n = 2692)	Random Forest	89.5	88.3	90.7	19.6	15.6	24.3	99.6	99.3	99.8
2023 Cohort	Decision Tree	52.1	44.8	59.4	4.2	1.2	10.4	97.1	91.6	99.4
(n = 197)	Random Forest	62.1	54.8	69.0	6.5	2.1	14.5	98.3	94.1	99.8

All models were trained on the subsampled training set with a case control ratio of 1:3. The models were then run to predict positive blood cultures in the subsampled test dataset (case control ratio 1:3), the full dataset, and the 2023 dataset. 95% confidence intervals (CIs) are given for all parameters

**Fig. 4:**
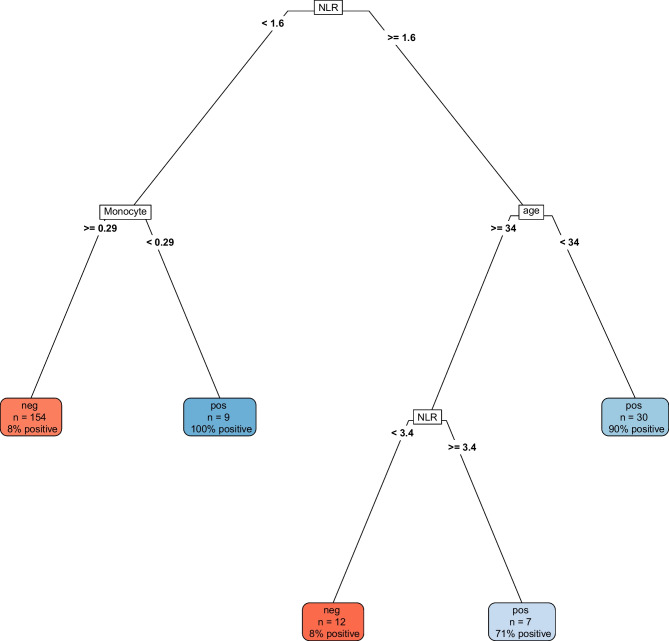
Decision tree showing prediction of blood culture results for infants aged 7–60 days assessed for suspected bloodstream infection at Children’s Health Ireland Temple Street, Dublin between 2005 and 2022. The decision tree is trained on the subsampled training dataset, containing 55 cases and 157 controls. The variables shown in the tree include neutrophile to lymphocyte ratio (NLR), monocytes (X10^9^/L) and age (days). Each leaf shows the predicted blood culture result according to the model (i.e.negative (neg) or positive (pos)), as well as the number of observations that fall within each leaf and the percentage of actual positive blood cultures in each leaf

**Fig. 5:**
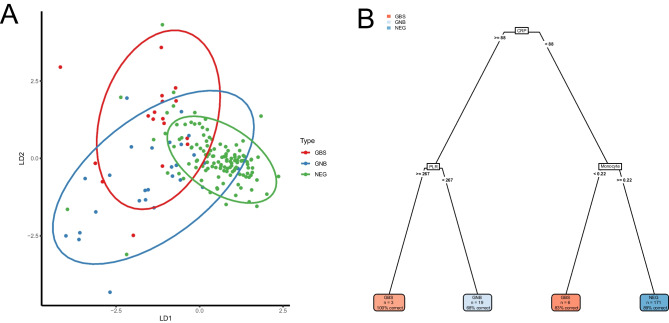
Segregation of Gram-negative bacilli (GNB), group B streptococcus (GBS), and negative cultures (NEG) for infants aged 7–60 days assessed for suspected blood stream infection at Children’s Health Ireland, location Temple Street, Dublin, between 2005 and 2022. Segregation is analysed by linear discriminant analysis (LDA) (**A**) and decision tree (**B**) using the subsampled training dataset, containing 55 cases and 157 controls. LD1 and LD2 in figure **A** refer to the axes constructed in the LDA analysis. In figure **B**, variables and cut-off values are shown in the decision tree, with C-reactive protein (CRP) in mg/L, monocytes in 10^9^/L and platelets to lymphocytes ratio (PLR). Each leaf shows the predicted outcome according to the model, as well as the number of observations that fall within each leaf and the percentage of correct predictions in each leaf

The decision tree model (Fig. 5) shows a root split based on NLR, and lower-level splits for monocytes, age and NLR. The largest bin in this group contains 73% of the study population, of whom 92% is culture negative. Another low-risk group consists of those with NLR ≥ 1.6 and < 3.4, and age ≥ 34 days, comprising 6% of the dataset. Simultaneously, we can see three high risk groups, those with NLR < 1.6 and monocytes < 0.29 × 10^9^/L (4%), those with NLR ≥ 1.6 and age < 34 days (14%) and those with NLR ≥ 3.4 and age ≥ 34 days (3%) (Fig. 4).

### Segregation of pathogen groups

LDA analysis on the distinct groups of pathogens showed good segregation of GBS and GNB versus controls (Fig. 5a). The variables contributing most to segregation of pathogen groups were CRP, MLR, PLR and neutrophils.

A single tree model on the distinct groups of pathogens showed good segregation of GBS and GNB versus controls in the training set (Fig. 5b). In the test set, it showed an overall accuracy of 89%. The variables that the segregation was based on were CRP, PLR, and monocytes.

### Application of the decision tree to the full dataset

When applied to the full dataset, the single tree model classified a total of 2342 (87%) of the children as low-risk, with a high negative predictive value of 99.1% (95% CI 98.6% – 99.4%). It classified a total of 350 infants as high-risk, with a positive predictive value of 15.4% (95% CI 11.8% – 19.6%). The overall accuracy remained high at 88.2%, while the AUROC dropped to 0.57.

The false-negative group contained 22 infants, of which four with GBS (n = 25 in the entire dataset), 12 with GNB (n = 36 in the entire dataset), four with *Staphylococcus aureus* (n = 6 in the entire dataset), one with *Haemophilus influenzae* and one with *Enterococcus faecalis*. The false positive group contained 296 infants, for whom we have no further clinical details.

When applied to the full dataset, the accuracy of the random forest model remained high at 89.5% while the AUROC dropped to 0.60. The model classified 12.7% of the infants as high risk, with a PPV of 19.6%. It classified 87.3% of the infants as low risk, with an NPV of 99.6%. Of the nine infants that were false negative, six had a blood culture positive for *Escherichia coli*, two for GBS and one for *Staphylococcus aureus*.

### Application of the models to the 2023 dataset

For six infants in the 2023 cohort, the blood culture grew a likely contaminant, and they were excluded from the dataset. The remaining dataset contained 197 infants, of whom seven had a positive blood culture. The pathogens cultured in this cohort were *Enterococcus faecalis* (n = 1), *Escherichia coli* (n = 3), *Staphylococcus aureus* (n = 1) and *Streptococcus agalactiae* (n = 2). The decision tree model classified 95 (48%) infants as high-risk with a PPV of 4.2%. It classified 102 (52%) infants as low-risk, with a NPV of 97.1%. The random forest model classified 77 (39%) infants as high-risk, with a PPV of 6.5%. It classified 120 (61%) children as low risk, with a NPV of 98.3% (Table 2). The overall accuracies (52.3% and 62.4%) and the AUROC (0.58 and 0.59) were low for both the decision tree and random forest model respectively.

## Discussion

In this study, we aimed to derive an easy-to-apply algorithm that can reliably identify infants aged 7–60 days with a low risk of positive blood cultures (and, by extension, BSI) presenting to an Emergency Department in a tertiary paediatric hospital setting in Ireland. This specific age group was chosen as the normal ranges of biomarkers and FBC parameters – which fluctuate after birth and during the first months of life – are rather stable in this age group.

All models tested in our study predicted positive blood cultures with similar sensitivity, specificity, NPV and PPV in a subsampled dataset with a case control ratio of 1:3. However, in the absence of dedicated decision support tools, implementation of these models would not be easy to establish in clinical practice. The decision tree model is an exception to this, as it makes use of a set of concrete cut-off values and can thus be interpreted at the bedside. We therefore selected the decision tree model for testing on the full dataset and the 2023 dataset, as well as the random forest model for comparison.

Both the decision tree model and random forest model showed to be good predictors for positive blood cultures in the full dataset with a high NPV, but low PPV, though the AUROC was rather low. In the 2023 cohort, the predictive value of both models was slightly lower. However, the NPVs were still high at 97.1% and 98.3% respectively. This is important, as an ideal predictive model would have a high NPV, approaching 100% [11]. Both models show low PPVs, under 10%. The low PPV and high NPV are in line with findings of other reports of models predicting positive blood cultures in both infant and adult populations. Despite a low PPV, these models are thought to be able to make a valuable contribution to the clinical decision-making process [12–14].

Though our models approach an NPV of 100%, they may not be able to identify all infants with BSI; some infants may be at an early stage of their infection, and FBC and biochemical parameters may not have shifted much at that point in time. The low PPV of our models may be due to infants in our cohort having similar clinical syndromes, such as localised infections not resulting in BSI, or viraemia. Additionally, a portion of the infants may have been diagnosed with culture negative sepsis [24], or a blood culture taken later in the course of disease might have come up positive. As we did not check for culture results from other sample types or follow up cultures, we do not know how much these scenarios have influenced our models’ parameters.

Ideally, a model as the ones presented in this study would have clinical parameters – e.g. temperature and heart rate – incorporated in addition to the laboratory parameters. Unfortunately, we were at present unable to incorporate such clinical parameters. Furthermore, we were unable to compare the prediction of the models to the judgement of physicians, as we did not have information on the initiated treatment. However, models like the ones presented in this study should never be used to solely base clinical decision making on. They should instead be used by physicians as a tool to further support clinical decision making, in addition to clinical expertise. The models presented in this study will therefore mainly be of benefit to clinical decision-making in infants without a clear indication for start of antimicrobial therapy, such as infants who do not appear particularly unwell, or who do not show clear signs of an infection source. In these cases, the models presented here can further support a low risk of BSI, and an observational approach without immediate start of treatment or further invasive procedures can be considered until blood culture results are available.

Furthermore, we have shown that different pathogen groups may induce different patterns in FBC and CRP shifts. Therefore, changes in circulation of pathogens over time could influence the accuracy of a model. During and after the SARS-CoV-2 pandemic, we have seen changes in circulation of many pathogens [25, 26]. This could further explain why our models, trained on data collected before and during the pandemic, underperform in predicting bloodstream infections in a post-pandemic dataset.

In conclusion, we have shown that both a random forest model and decision tree model, trained on data from 2005–2022 in a tertiary hospital in Dublin, Ireland, can predict positive blood cultures in infants aged 7 to 60 days with a high NPV. While the random forest model performs slightly better, the decision tree model is easy to implement and could be of direct assistance in clinical practice. While the PPV of these models is low, these models can support practicing clinicians in recognizing low-risk patients, for whom a reserved attitude towards starting treatment and further diagnostic procedures may be appropriate.

A future validation study is necessary to specifically assess children who do not have clear clinical indication for starting therapy, such as infants who appear particularly unwell or those with a clear source of infection. In addition, further development of these models is desirable to increase the PPV, thereby reducing the number of false positives. Separate models that predict positive blood cultures for distinct pathogen groups may prove more accurate compared to a single model predicting overall positive blood cultures as presented here. Furthermore, it will be important to continuously train these models, to ensure that they remain applicable in changing microbiological landscapes. Lastly, as the models presented here were trained on infants aged 7 to 60 days presenting at the emergency department, the application of these models is limited to infants in this age range with suspected community acquired BSI, and may not be applicable to any other patient groups.

### Supplementary Information

Below is the link to the electronic supplementary material.

Supplementary file1 (DOCX 12 KB)

## Data Availability

Clinical data used for this study were extracted from the electronic laboratory information system at CHI Temple Street, Dublin, Ireland. This data is not publicly available to maintain patients’ privacy in line with the European General Data Protection Regulation. Anonymized data is available on request.
