# Supplementary material for: Predicting community acquired bloodstream infection in infants using full blood count parameters and C-reactive protein; a machine learning study
Source: Eur J Pediatr. 2024 Apr 18;183(7):2983–93. doi: 10.1007/s00431-024-05441-6 (PMC11192659; doi:10.1007/s00431-024-05441-6)
Supplement: Supplementary file 1 — Supplementary file1 (DOCX 12 KB) [file 431_2024_5441_MOESM1_ESM.docx]

*Staphylococcus hominis
Staphylococcus capitis
Staphylococcus epidermidis
Staphylococcus simulans
Staphylococcus haemolyticus
Staphylococcus auricularis
Staphylococcus saprophyticus
Staphylococcus warneri
Streptococcus salivarius
Streptococcus viridans
Acinetobacter lwoffi
Acinetobacter baumanii
Neisseria sicca
Pantoea agglomerans
Gemella* species
Diphteroids *Kocuria* species *Micrococcus luteus
Peribacillus simplex
Streptococcus parasanguinis
Streptococcus mitis/oralis*
